# Use It or Lose It: Environmental Enrichment as a Means to Promote Successful Cognitive Aging

**DOI:** 10.1100/tsw.2010.111

**Published:** 2010-06-16

**Authors:** Karyn M. Frick, Jamie D. Benoit

**Affiliations:** ^1^ Department of Psychology, Yale University, New Haven, CT, USA; ^2^ Department of Psychology, University of Wisconsin, Milwaukee, USA

**Keywords:** hippocampus, memory, exercise, cognitive stimulation, rodent

## Abstract

Environmental enrichment has become increasingly utilized in rodent models of aging and neurodegenerative disease in order to prevent or reverse cognitive decline and neuronal dysfunction. However, the potential application of this body of work to human cognitive aging has rarely been discussed. The present article provides an overview of the rodent research that has tested the effects of environmental enrichment on hippocampal and neocortical function, and the types of memories mediated by these brain regions. Although data from models of neurodegenerative disease are presented, primary emphasis is given to studies of aging rodents and to methodological issues (e.g., age, treatment duration, treatment type) central to the mnemonic effectiveness of enrichment treatment. The implications of this work for human cognitive aging are discussed.

## INTRODUCTION

Regions of the brain that are critical for cognitive function, such as the hippocampus and neocortex, are especially vulnerable to the detrimental effects of aging[1,2,3]. Hippocampal and neocortical dysfunction is characteristic of normal aging (e.g., [4]) and leads to a variety of cognitive impairments, including deficits in spatial navigation, object recognition, working memory (i.e., short-term), planning, and executive function[5,6,7,8]. Severe deterioration of both brain regions is a hallmark of Alzheimer's disease and other dementias[9,10,11]. With ever-increasing numbers of people living over the age of 65 in the U.S. and other industrialized nations, the need to promote successful cognitive aging has never been more important. Indeed, the number of people in the U.S. age 65 and older with Alzheimer's disease is expected to increase 50% (to nearly 8 million) by the year 2030[12]. In just 2005 alone, the costs to Medicare and Medicaid for the care of patients with dementia and to businesses for lost productivity by caregivers topped over $148 billion[12]. These costs will skyrocket in the coming decades as the Baby Boomers surpass 65 years of age. Because age is the primary risk factor for dementia[13], it is increasingly important to preserve cognitive and neural functioning through very advanced ages.

Behavioral treatments, including cognitive stimulation and physical exercise, may be especially useful for cognition enhancement in the elderly because most elderly already take multiple prescription drugs for conditions such as hypertension and high cholesterol[14]. Many of these medications can negatively affect cognition on their own[15] and the use of multiple medications can lead to drug interactions that have unintended consequences for cognitive function. In contrast, cognitive stimulation (e.g., extensive reading, travel, continuing education, participation in clubs and professional organizations) and physical exercise (e.g., walking, gardening, dancing, tennis, jogging) may provide significant benefits to overall health and cognitive function without risking drug interactions, and these treatments are simple to implement at any age. Although a focus on behavioral change may appear trivial in the face of the devastating neuropathological sequelae of aging and dementing illnesses, emerging evidence strongly suggests that cognitive stimulation and physical exercise can reduce the detrimental effects of aging and neurodegenerative disease on cognitive function. For example, cognitively stimulating occupations (e.g., university professor) can prevent age-related decline in verbal and working memory relative to the general aging population[16], and greater levels of education are strongly associated with reduced risk of age-related cognitive decline, dementia, and Alzheimer's disease[13,17]. More recent data show that cognitively stimulating leisure activities (e.g., reading, crosswords, organized group discussions, cultural events) late in life can reduce the risk of dementia[18,19] and delay the onset of memory decline in healthy elderly persons who later develop dementia[20]. Physical exercise (e.g., walking, stair climbing, recreational activities) has also been associated with reduced global cognitive decline[21,22,23] and increased neocortical and hippocampal volume[24,25,26] in elderly men and women, even when this treatment is started later in life. Although these data are compelling, relatively few studies have been conducted in humans, and the ethical constraints inherent to work with humans prevent an understanding of the cellular and molecular mechanisms in the brain that underlie the cognitive benefits of enriching stimulation. As such, animal models can be extremely useful in order to understand the potential of cognitive and physical stimulation to prevent or reduce age-related cognitive decline. The vast majority of such work to date has been conducted in rodents, where most studies have examined effects of cognitive and physical stimulation on memory mediated by the hippocampus and neocortex. These brain regions regulate many types of memory that decline with age in both humans and rodents, including spatial memory (e.g., used to navigate through our environments), and memory for objects and odors[6,7,8,27,28]. Given the similarity of age-related cognitive and neurobiological decline in rodents and humans, this review will focus on research conducted in rodents that tested the effects of cognitive and physical stimulation on hippocampal and neocortical function, and the types of memories (e.g., spatial, object, working, contextual) subserved by these brain regions. Although data from young rodents and models of neurodegenerative disease will be presented, primary emphasis will be given to studies of aging rodents and methodological issues (e.g., age, treatment duration, treatment type) central to the effectiveness of treatment. Finally, the implications of this work for human cognitive aging will be addressed.

## ENVIRONMENTAL ENRICHMENT AS A MODEL OF COGNITIVE AND PHYSICAL STIMULATION

The effects of cognitive and physical stimulation on the brain and behavior are modeled in rodents using a paradigm called environmental enrichment. Environmentally enriched rodents are typically socially housed in large groups and exposed to a variety of stimulus objects that can provide both cognitive stimulation (e.g., toys, tunnels, dwellings) and physical exercise (e.g., running wheels). Enrichment can take place in the home cage (standard size or larger)[29,30,31,32] or for several hours each day outside of the home cage[33,34,35]. Control animals are housed in standard laboratory cages, either individually (isolated controls) or in small groups (social controls). Most early work on enrichment, dating from the 1960s and 1970s, examined differences between controls and young adult rodents raised in enriched environments from weaning. These studies demonstrated profound effects on regions of the brain critical to learning and memory, such as the neocortex and hippocampus. For example, in the neocortex, enrichment increased cortical thickness, dendritic spines and branching, synaptic contacts and neurotransmission, and neuron size[36,37,38,39,40,41,42,43,44,45]. Enriched rodents also exhibited enhanced learning and memory abilities relative to control littermates[45,46,47,48,49].

Subsequent work showed that the beneficial effects of environmental enrichment on memory and brain function could also be produced when enrichment is initiated in young adulthood (typically 3–6 months of age). In the neocortex of rats and mice enriched from young adulthood, treatment increased levels of several neurotrophic factors[32,50] and the expression of genes related to neuronal signaling and growth, RNA expression, and protein synthesis and processing[35]. In the hippocampus, a brain region particularly involved in regulating spatial learning and memory, enrichment in adulthood enhanced long-term potentiation[51], synaptic strength[52,53], and neurogenesis[31,54], and increased levels of growth factors and synaptic proteins[32,50,55,56]. Accordingly, many of these hippocampal alterations, including changes in long-term potentiation, neurogenesis, and growth factors, were associated with enrichment-induced improvement in hippocampal-dependent spatial and contextual memory[51,54,55,57].

Other work in young adult rodents has demonstrated that enrichment can promote recovery from genetically induced deficits in the hippocampus. For example, in mice with a deletion of the gene for a specific NMDA receptor subunit (NR1) in the CA1 region of the hippocampus, 3 h of enrichment per day for 2 months significantly attenuated knockout-induced deficits in object recognition, contextual fear, and olfactory discrimination memory[34]. Enrichment also increased CA1 dendritic spine density in NR1 knockouts, suggesting that experience-induced alterations in spine density can occur in the absence of NMDA receptor activity[34]. However, 3 h of enrichment per day for just 2 weeks significantly increased protein levels in the hippocampus of several glutamate receptor subunits, including GluR1, NR2A, and NR2B (but not NR1) in wild-type and NR2B-overexpressing mice, suggesting that enrichment can affect both hippocampal AMPA and NMDA receptors[58]. Enrichment can also ameliorate memory and synaptic plasticity deficits in mice with induced overexpression of the protein p25[59], which has been implicated in Alzheimer’s disease and which causes neurodegeneration, tau hyperphosphorylation, and increased amyloid-beta secretion[60]. In this mouse model, p25 overexpression was induced for 6 weeks prior to enrichment, leading to brain atrophy and impairments in spatial memory and contextual fear conditioning[59]. Four weeks of subsequent enrichment had no effect on general brain atrophy, but did significantly increase hippocampal levels of synaptic proteins and re-establish access to long-term spatial and contextual fear memories[59], suggesting that the beneficial effects of enrichment on memory stemmed mainly from enhanced synaptic plasticity rather than a slowing of general brain atrophy. Enrichment also increased acetylation and methylation of histone proteins H3 and H4 in the hippocampus and neocortex of p25 transgenics, suggesting that epigenetic effects on chromatin remodeling may play a role in the beneficial effects of enrichment on memory[59].

Collectively, the data from young adult rodents suggests that enrichment initiated in adulthood produces a multifaceted response in the hippocampus and neocortex that leads to increased synaptic plasticity and enhancement of several types of memory. Moreover, enrichment treatment can protect against the detrimental effects of several genetic alterations that compromise hippocampal function, suggesting that interactions with enriching stimuli induce robust changes in the hippocampus that promote recovery of function.

## ENRICHMENT IN AGING RODENTS

The potential neuroprotective effects of enrichment are of great relevance to cognitive aging, and numerous studies have examined the effects of enrichment on memory and hippocampal function in rodents that are middle aged (approximately 14–19 months old) and aged (20 months and older). Among middle-aged rodents, environmental enrichment initiated at weaning or during middle age increases dendritic branching, forebrain weight, and neurotrophin levels in the neocortex[61,62,63,64], and increases neurogenesis, synaptic vesicle number, and neurotrophin levels in the hippocampus[63,64]. Although only a handful of behavioral studies have been conducted in middle-aged rodents to date, the available data suggest that the beneficial effects of enrichment on learning and memory are similar in nature to those in young rodents. For example, several studies report that enrichment in middle-aged mice and rats improves spatial memory in the Morris water maze relative to socially housed nonenriched middle-aged controls[29,63,65,66]. In the water maze, rodents must find an escape platform submerged just underneath the surface of a large round tank of water. Because the platform location is hidden from view, subjects must navigate to it using spatial cues outside of the maze. Studies of enriched middle-aged rodents report that these subjects are significantly better at finding the platform location than nonenriched subjects, and often perform as well as young controls[29,63,65,66]. Enrichment-induced improvements in middle-aged rodents have been observed when enrichment is initiated at weaning[55], in young adulthood[67,68], or in middle age[29,65], suggesting that treatment can both prevent and reverse age-related memory decline. Further, similar effects have been reported in males and females[29], indicating that both sexes can benefit from treatment. A recent study of middle-aged female mice also found that lifelong enrichment enhanced both novel object recognition and spatial memory[66], which suggests that enrichment can affect multiple types of memory in middle-aged rodents. The enrichment-induced improvements in spatial memory among middle-aged rodents have been associated with alterations in several brain regions, including enhanced neurogenesis in the hippocampus[65]; increased nerve growth factor (NGF) levels[67] in the hippocampus, visual cortex, and entorhinal cortex; and increased NGF receptors in the medial septum[69]. These data suggest that various alterations in the hippocampus and its cortical and subcortical afferents may be integral to the beneficial effects of enrichment on memory in middle-aged rodents.

Among aged rodents, enrichment initiated at weaning, in young adulthood, or during old age can improve several types of learning and memory, including spatial memory in the Morris water maze[29,30,33,65,69,70,71] and novel object recognition[66,68,72]. Enrichment also reverses short-term memory deficits, increases spontaneous alternation in a Y maze, and increases food-seeking behaviors[73,74,75,76]. However, the behavioral benefits are not universal; enrichment in aged male rodents does not increase reactivity to spatial novelty or general locomotor activity, nor does it improve performance in the Lashley III maze or a brightness discrimination[74,75]. In the brains of aged rodents, enrichment has numerous effects in several regions, including increased Purkinje cell dendritic branching in the cerebellum[77] and increased cholinergic neuron number in the basal forebrain[69]. In the neocortex, enrichment has been shown to increase spine density, RNA content, and presynaptic vesicle number[75,78,79,80]. In the hippocampus, enrichment reduces gliosis[73] and increases dendritic branching and spine density in the dentate gyrus[81]. In both the neocortex and hippocampus of aged rodents, enrichment also increases levels of the presynaptic protein synaptophysin[33,82]. Several of these enrichment-induced alterations have been associated with improved memory function. For example, enrichment-induced improvements of spatial memory in the Morris water maze and of novel object recognition have been associated with increased hippocampal neurogenesis and synaptophysin levels in aged female mice[33,65,68]. Interestingly, one recent study of aged male rats showed that enrichment need not be administered constantly to benefit memory and hippocampal function; beginning at 3 months of age, 18 months of exposure to an enriched environment for only 3 h/week significantly enhanced novel object recognition and increased hippocampal neurogenesis and synaptophysin levels[68]. Together, these studies demonstrate that environmental enrichment can produce substantial and wide-ranging improvements in both memory and neural function in aged rodents, and suggest considerable capacity for plasticity in the aged brain.

## ENRICHMENT TREATMENT: ONE TYPE FITS ALL?

Although the above review of the enrichment literature may suggest that enrichment benefits memory at any age, studies that have directly compared the effects of environmental enrichment on memory in young, middle-aged, and aged rodents demonstrate that the effects of a given enrichment treatment on memory can depend on age. For example, in one study of female mice, exposure for 4 weeks to enrichment in the home cage improved novel object recognition in aged (21 months old) females, but not young (5 months old) females[72]. Similarly, in another study of male mice exposed to the same enrichment treatment for 6 weeks, spatial memory in the Morris water maze was improved in aged (21 months old) males, but not in young (3 months old) or middle-aged (15 months old) males[70]. Although these data might suggest that young and middle-aged mice do not respond to short-term enrichment, other data show that 4 weeks of a less-complex home-cage enrichment treatment can modestly improve spatial Morris water maze performance in middle-aged male and female mice[29]. Furthermore, lifelong enrichment appears to provide clear benefits for middle-aged mice. Indeed, among female mice, home cage enrichment initiated at weaning improved spatial Morris water maze performance in middle-aged (17 months old) and aged (22 months old) females, but not in young (5 months old) females[66]. In this same study, novel object recognition memory was also improved in middle-aged and aged females, but not in young females[66], suggesting similar effects of enrichment on multiple types of memory in middle and advanced age.

These findings beg the question of whether the specific enrichment protocol used influences the ability of enrichment to affect memory or brain function. In fact, there is a wide disparity among enrichment paradigms used by different laboratories, which makes it difficult to evaluate the effects of enrichment on memory and the brain. Enrichment treatments in the literature vary in a number of ways, including cage size, number of cagemates, stimulus complexity, total duration of treatment, duration of treatment per day, frequency with which novel stimuli are introduced, amount of experimenter handling, inclusion of supplemental food treats, and the control group to which enriched animals are compared (i.e., social or isolated)[30]. Few studies have systematically tested the effects of these factors on memory and neural function at any age, so it is unclear if any of these variables might substantially influence experimental outcomes. However, several recent studies have begun to address these issues in aging rodents as discussed below.

One study of aged male mice compared two commonly used enrichment procedures: 24-h/day enrichment in a large home cage (termed “continuous enrichment”) and 3-h/day enrichment in a cage separate from the home cage (termed “daily enrichment”)[30]. Both types of enrichment can be effective; continuous enrichment improves memory and enhances hippocampal and neocortical plasticity in a host of studies of young and aging rodents (e.g., [31,32,45,51,54,63,64,65,70,71]), whereas daily enrichment improves spatial memory in aged females[33] and protects young females against the memory-impairing effects of NMDA NR1 gene knockout[34]. In one study, aged male mice were group housed for a total of 10 weeks in standard shoebox cages or in large cages with many cagemates and a variety of rodent toys and running wheels (continuous enrichment; Fig. 1A). Aged mice housed in standard shoebox cages received no treatment (control), daily handling, and exposure to an empty novel cage (daily handling), or placement with cagemates in a medium-sized cage containing a running wheel and a few rodent toys for 3 h/day (daily enrichment). All three aged groups were compared to a group of young control mice that were group housed in standard shoebox caging. Six weeks after the start of enrichment, spatial reference and working memory were tested in the standard Morris water maze task and in a water-escape motivated radial arm maze. Daily handling had no effect on either spatial task (Fig. 1B; [30]), suggesting that daily experience with handling and novel caging does not contribute to the mnemonic effects of enrichment. Daily enrichment also had no effect on memory in either task (Fig. 1B; [30]), which is inconsistent with the beneficial effects of this treatment on spatial memory in aged female mice[33]. In contrast to daily enrichment, continuous enrichment significantly improved spatial memory in both tasks, such that the performance of aged continuously enriched males was statistically indistinguishable from that of young males (Fig. 1B; [30]). Interestingly, this improvement was associated with a decrease in synaptophysin levels in the hippocampus and frontoparietal cortex[30], suggesting a potentially negative relationship between enrichment-induced alterations in spatial memory and synaptophysin levels in aged males. This apparent negative relationship is contrary to the positive relationship observed between spatial memory and synaptophysin in enriched aged females[33]. Indeed, sex differences in this relationship, in addition to the differential effectiveness of daily enrichment in aged males[30] and females[33], suggest potentially important differences in the effects of certain enrichment protocols that may result from sex

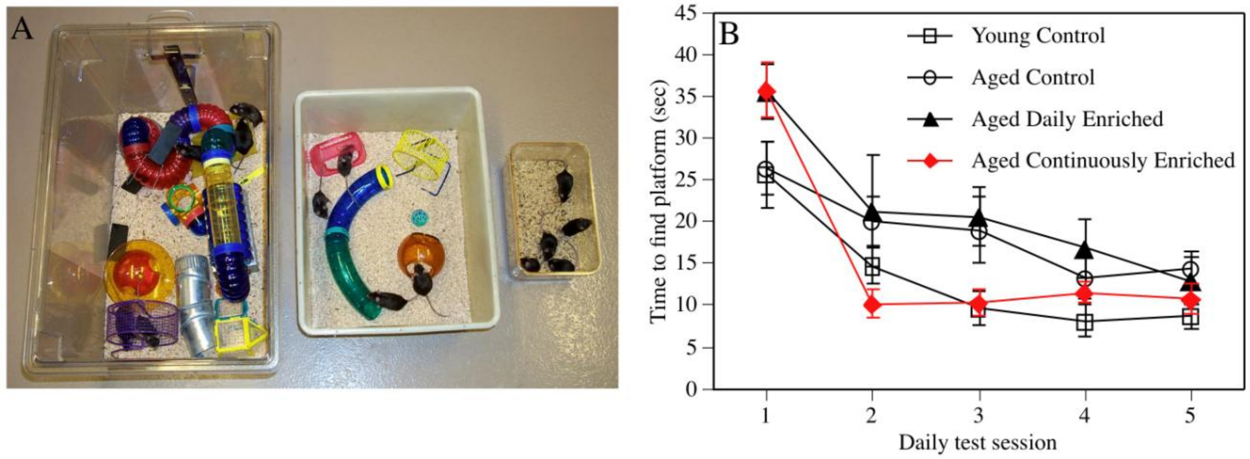

**FIGURE 1**. (A) Photograph of the enrichment conditions used by Bennett et al.[30]. Continuous enrichment is shown at left, a bin used in the daily enrichment condition is shown in the middle, and standard housing used for all groups but the aged continuously enriched group is shown at right. (B) Spatial memory in the Morris water maze as illustrated by time to find the hidden escape platform. The aged control group includes mice that received daily handling, as these mice did not differ statistically from nonenriched controls. Only aged continuously enriched mice were unimpaired relative to young controls. Each point represents the mean ± standard error of the mean (SEM) of each group during one five-trial test session. Figure modified from Bennett et al.[30].

differences in locomotor activity, motivation to explore, or the nature of affected synapses[30]. Nevertheless, within aged males, there were several obvious differences between the daily enrichment and continuous enrichment conditions that may have led to the discrepant effects of these treatments on spatial memory[30]. These differences include stimulus object number and complexity, and time spent with the objects per day. Another critical difference is the time of day in which mice were exposed to the objects. Daily enrichment occurred during the light phase of the cycle only, whereas continuous enrichment also allowed mice to interact with stimuli during the dark phase of the cycle, when they are most active. Thus, mice may have interacted more with the stimuli in the continuous enrichment condition because they could do so at a time of day when they were most apt to explore[30]. Although this study did not pinpoint which of these differences contributed most to the discrepant effects of daily and continuous enrichment, the findings highlight the fact that different types of enrichment can have discrepant effects on memory on rodents of the same age, and suggest that greater attention be paid to understanding the elements of the enriched environment that are critical for reaping the beneficial effects of this treatment on cognition.

## COGNITIVE STIMULATION VS. EXERCISE

Along these lines, one area ripe for investigation is determining whether cognitive stimulation or physical exercise differ in their ability to enhance memory function.

In considering the application of enrichment treatment to humans, it must be acknowledged that maintaining a healthy and active lifestyle can be challenging. This can be especially true after retirement, when health or logistical obstacles may prevent the elderly from maintaining adequate cognitive and physical stimulation. Although the few clinical studies conducted on this topic to date suggest that education, cognitive stimulation, and physical exercise can individually improve cognition in the elderly[16,18,19,20,21,22,23], cognitive stimulation and physical exercise have never been directly compared to determine their relative effectiveness in promoting successful cognitive aging. In part, performing this comparison in humans can be difficult because most data are obtained from subjective self-reports, rather than clinical trials of specific treatment regimens. Nevertheless, this information could be critically important for designing behavioral interventions for the elderly and lifestyle recommendations for younger people. That is, if cognitive stimulation and physical exercise differ in their ability to prevent age-related cognitive decline, then interventions could be more specifically tailored towards the most effective treatment in order to provide the greatest benefits for cognitive health.

In rodents, voluntary physical exercise alone can produce many of the benefits of environmental enrichment. For example, voluntary exercise increases hippocampal synaptophysin levels, growth factor levels, cholinergic receptor density, protein kinase C binding, long-term potentiation, and neurogenesis[83,84,85,86,87]. Exercise also increases angiogenesis in the neocortex, hippocampus, and cerebellum[87,88,89,90], providing much-needed nutrients and energy to the brain, including glucose and oxygen. In elderly humans, 6 months of exercise treatments consisting of at least three 50-min aerobic exercise sessions per week significantly enhanced global cognitive function[23] and increased the volume of the anterior cingulate cortex, supplementary motor cortex, right inferior frontal gyrus, and left superior temporal gyrus[24]. In rodents, voluntary exercise improves spatial memory in several tasks including the Morris water maze, a place-learning set task, and the radial arm maze[85,86,91]. These data suggest that exercise is a critical component of the enrichment experience in both humans and rodents.

Indeed, two recent papers from our laboratory demonstrated that this might be so, particularly for young adult and middle-aged female rodents. In one study of young female mice, exposure for 6 weeks (3 h/day) to running wheels, but not to cognitively stimulating toys or training in an acrobatic course, significantly improved spatial working memory in a water escape–motivated radial arm maze[92]. Although this finding suggests that voluntary exercise is more critical in young females to improving spatial memory than cognitive stimulation, it is possible that 3 h/day were simply not enough to reap the benefits of cognitive stimulation. Therefore, a follow-up study exposed mice to exercise or cognitive stimulation for 24 h/day in large home cages[71]. To better compare the effects of each treatment to more standard enrichment protocols, a “complex enrichment” treatment was included that provided both exercise and cognitive stimulation (Fig. 2). Finally, to determine whether the effects of these treatments differed across the lifespan, young (3 months old), middle-aged (15 months old), and aged (21 months old) females were tested using a cross-sectional design. Among young females, spatial memory in the Morris water maze was enhanced only by exercise alone (Fig. 2B; [71]). Among middle-aged females, both exercise alone and complex enrichment, but not cognitive stimulation, improved spatial memory (Fig. 2B; [71]). Together, these data suggest that exercise is the key component necessary for environmental enrichment to enhance spatial memory in young and middle-aged female mice. In contrast, all enrichment treatments, including cognitive stimulation, improved spatial memory in aged females (Fig. 2B; [71]), indicating that either exercise or cognitive stimulation can improve memory in aged subjects. Several important conclusions may be drawn from this study. The first is that different elements of the enriched environment can have discrepant effects on memory at different points in the lifespan. This important point illustrates that type of enrichment should be carefully considered when planning enrichment studies in multiple age groups. The second conclusion is that exercise alone can improve memory at any age, which pinpoints exercise as a key aspect of the enrichment treatment. The general effectiveness of exercise may be due to its ability to enhance both synaptic plasticity and angiogenesis in the hippocampus and neocortex[89]. Finally, the data indicate that almost any type of enrichment can improve memory in aged subjects, which is encouraging for the application of behavioral treatment strategies for reducing cognitive decline in humans.

## ENRICHMENT IN MODELS OF NEURODEGENERATIVE DISEASE

In addition to normal aging, environmental enrichment has been increasingly utilized in rodent models of several neurodegenerative diseases, including Alzheimer’s, Parkinson’s, and Huntington’s diseases (see [93] for recent review). For example, in transgenic mouse models of Huntington’s disease, a hereditary neurodegenerative disease that impairs movement, executive function, memory, and emotion, wheel running or complex enrichment reduces abnormal motor behaviors, improves spatial memory, increases hippocampal and neocortical synaptophysin levels, and delays the onset of deficits in postsynaptic

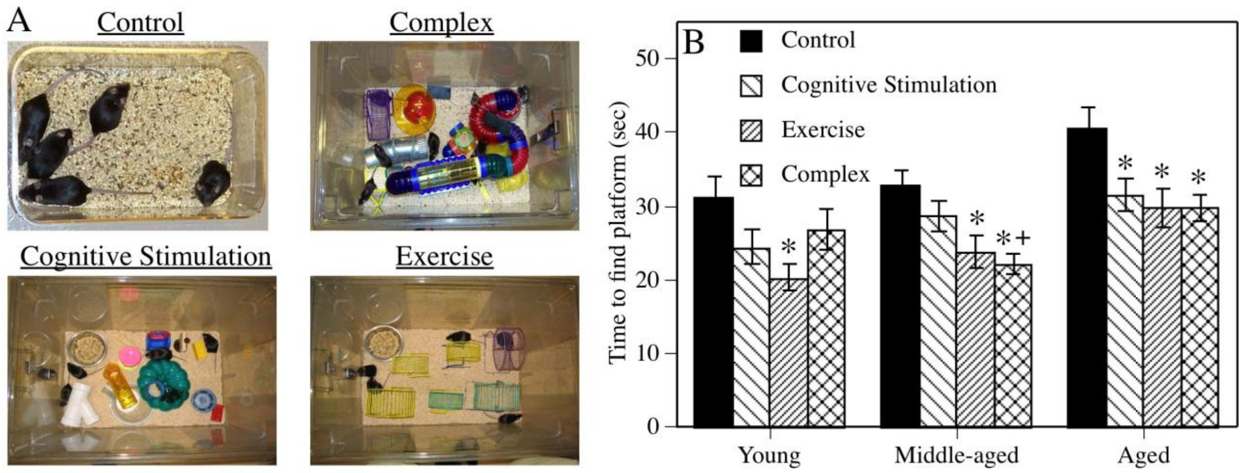

**FIGURE 2**. (A) Photographs of the enrichment conditions used by Harburger et al.[71]. (B) Spatial memory in the Morris water maze as illustrated by time to find the hidden escape platform. Each bar represents the mean ± SEM of five daily test sessions. * = significant difference from same-age controls, + = significant difference from same-age cognitive stimulation group. Figure modified from Harburger et al.[71].

plasticity[94,95]. In a pharmacological model of Parkinson's disease, enriched mice showed significantly less dopaminergic neuron loss in the substantia nigra after treatment with 1-methyl-4-phenyl-1,2,3,6-tetrahydropyridine (MPTP), a neurotoxin that induces Parkinson's-like motoric dysfunction in humans[96].

However, the vast majority of studies on enrichment and neurodegenerative disease have been conducted using transgenic mouse models of Alzheimer's disease. Studies of various Alzheimer's transgenics report numerous beneficial effects of enrichment in the brain, including increased cerebral angiogenesis, and reduced cerebral oxidative stress and apoptotic enzyme expression[97,98,99]. Enrichment in these mice also increases hippocampal neurogenesis, synaptophysin immunoreactivity, neurotrophin levels, and immediate early gene expression[97,100,101,102,103,104]. Other studies report that enrichment in Alzheimer's transgenics can improve spatial memory in tasks like the Morris water maze and radial arm maze[101,102,104,105,106,107], and one study found that superior spatial working memory in the radial arm maze was correlated with lower hippocampal amyloid-beta (Aβ) levels[102]. Indeed, a recent study of transgenic male mice coexpressing mutant human presenilin 1 (PS1ΔE9) and APPswe found that enrichment from weaning through 6 months of age significantly reduced amyloid deposition in the hippocampus and neocortex, and steady-state levels of Aβ peptides in the neocortex[103]. The reduction in amyloid deposition was inversely correlated with increased activity, such that enriched mice exhibiting the highest activity levels had less amyloid deposition than standard housed mice or enriched mice with low activity levels[103]. However, the effects of enrichment on amyloid plaque burden and Aβ levels are far from clear. Although most studies show no effect of enrichment on amyloid deposition or plaque burden[100,101,104,107], other studies find reductions in Aβ levels[102,103], whereas yet others report increased Aβ levels and plaque load[105,106]. Given that these studies used a variety of transgenic models, these inconsistencies are perhaps not entirely surprising. Collectively, however, studies of Alzheimer's transgenics do suggest that enrichment has beneficial effects on hippocampal memory and plasticity irrespective of effects on amyloid plaques and Aβ levels. This conclusion is further supported by the widespread effects of enrichment on gene expression in APPswe x PS1ΔE9 transgenics, where enrichment increased expression of genes associated with learning and memory, neurogenesis, cell survival, Aβ sequestration, and vasculogenesis[103]. Interestingly, the various effects of enrichment in Alzheimer's transgenics have only been observed after complex enrichment (i.e., cognitive stimulation plus exercise) and not after exercise alone; multiple transgenic studies have reported that complex enrichment, but not exercise alone, improves spatial memory, increases hippocampal neurogenesis and growth factor levels, and/or decreases Aβ levels[100,101,102]. The ineffectiveness of exercise is surprising in light of the studies in wild-type mice discussed above in which exercise was the critical component of enrichment at all ages[30,56]. As such, the transgenic data suggest that either cognitive stimulation or a combination of cognitive stimulation and exercise is required to protect against the neurodegenerative effects of amyloid precursor protein and presenilin overexpression. Future studies will need to address this issue.

Nevertheless, the data from Alzheimer's and other neurodegenerative disease models support the encouraging, if not surprising, possibility that the devastating mnemonic and neuropathological effects of several neurodegenerative diseases can be ameliorated by a treatment as simple as environmental enrichment. Indeed, studies in humans have shown that greater levels of education and cognitively stimulating leisure activities can reduce the risk of developing dementia and Alzheimer's disease[13,17,18,19]. Although the specific neurobiological mechanisms underlying this reduced risk are currently unclear, animal models such as those reviewed above will undoubtedly lead to a much greater understanding of the molecular and cellular processes that are involved.

## CONCLUSIONS

This review has focused on animal models of environmental enrichment as a means of understanding how cognitive stimulation and physical exercise can reduce age-related cognitive and neurobiological decline. However, the parallels to humans should not be overstated; although useful, animal models cannot fully reproduce the complicated interplay among education, social enrichment, physical exercise, and health care characteristic of human development. Further, it should be noted that animals housed in standard laboratory conditions live in environments that are quite impoverished relative to human environments and, thus, the effectiveness of enrichment treatments in animals may be greater relative to those in humans given the stimulation-deprived nature of the standard control group. Nevertheless, animal models provide more control over various environmental factors than is possible in humans, and so may allow for a greater insight into how specific elements of an enriched environment contribute to successful cognitive and neural aging.

If applicable to humans, the implications of the rodent data for human aging are clear. For normal aging, they suggest that physical exercise can enhance cognitive function throughout the lifespan, which could lead to stronger public health recommendations that promote the benefits of exercise for cognitive health in both youth and old age. For the elderly, such recommendations could lead to greater availability of organized exercise programs in senior centers and nursing facilities. For subjects at risk of neurodegenerative disease, the data from mouse models provides hope that cognitively and physically enriching activities might delay the onset of dementia or reduce its severity. The findings also indicate for normal aging that cognitive stimulation (e.g., continuing education, reading, chess, card games) late in life can be as beneficial for memory as exercise, which would be good news for elderly whose physical activity is restricted due to health problems. Especially encouraging, however, is the fact that enrichment started late in life can provide significant benefits for cognitive function. As such, it may not be necessary to maintain a physically or cognitively active lifestyle throughout life, although doing so clearly benefits all aspects of one's health. This conclusion may provide hope to people of all ages that it is never too late to get on track towards successful cognitive aging.

## ACKNOWLEDGMENTS

The authors gratefully acknowledge the support of Yale University and NIH grant R01 AG022525 to KMF. We also thank Dr. Lauren Harburger and Ms. Brianne Kent for their critical reading of this manuscript. Experimental work from the Frick laboratory discussed in this review was supported by grants from the Alzheimer's Association (IIRG-03-6051), NIH grants MH065460 and AG022525, the

American Federation for Aging Research/Pfizer, and the Claude D. Pepper Older Americans Independence Center at Yale University School of Medicine (P30AG21342).
